# Dpep, a Cell-Penetrating Peptide Targeting ATF5, CEBPB and CEBPD, Synergistically Combines with ABT-263 and Decitabine to Inhibit Cancer Cell Growth and Overcome Dpep Resistance

**DOI:** 10.3390/cells15090826

**Published:** 2026-05-01

**Authors:** Qing Zhou, Trang Thi Thu Nguyen, James M. Angelastro, Markus D. Siegelin, Lloyd A. Greene

**Affiliations:** 1 Department of Pathology and Cell Biology, Vagelos College of Physicians & Surgeons, Columbia University Irving Medical Center, New York, NY 10032, USA; qz2266@cumc.columbia.edu (Q.Z.); thithutrang.nguyen@nyulangone.org (T.T.T.N.); ms4169@cumc.columbia.edu (M.D.S.); 2 Ronald O. Perelman Department of Dermatology, Perlmutter Cancer Center, NYU Grossman School of Medicine, NYU Langone Health, New York, NY 10016, USA; 3 Department of Molecular Biosciences, School of Veterinary Medicine, University of California Davis, Davis, CA 95616, USA; jmangelastro@ucdavis.edu

**Keywords:** cancer, anti-cancer, cell-penetrating peptide, Dpep, Bpep, ABT-263, decitabine, overcoming resistance, apoptosis, combination therapy

## Abstract

**Highlights:**

**What are the main findings?**

- Dpep, a cell-penetrating peptide that targets ATF5, CEBPB and CEBPD, synergizes with BH3-mimetic ABT-263 and hypomethylating agent decitabine to kill cancer cells, including those selected for Dpep resistance.
- The combination of Dpep and ABT-263 shows a “cure rate” of 40% in a mouse melanoma xenograft model.

**What is the implication of the main finding?**

- Dpep has potential as a clinical treatment for a range of cancers, particularly as a component of an appropriate combination therapy.

**Abstract:**

Dpep is a cell-penetrating peptide that targets transcription factors ATF5, CEBPB and CEBPD to selectively suppress growth and survival of diverse tumor cell types in vitro and in vivo. Due to these actions and its apparent safety, the peptide has potential as a cancer therapeutic. How Dpep might be combined with other anti-cancer agents to achieve synergistic efficacy and to overcome possible peptide resistance has not been assessed in depth. Based on prior work indicating that Dpep promotes apoptotic cancer cell death and up-regulates multiple pro-apoptotic and tumor suppressor genes, we studied combinations of Dpep with ABT-263, a pro-apoptotic BCL2 family inhibitor, and decitabine, a hypomethylating drug. Combining Dpep with each agent alone or together synergistically suppressed the growth of a range of solid and liquid tumor cell types. Moreover, the combinations synergistically inhibited the growth of cells lines that were selected either in vivo or in vitro for Dpep resistance. Finally, we tested the combination of Dpep with ABT-263 in a mouse melanoma xenograft model. The combination more effectively inhibited tumor growth than either agent alone and, in contrast to vehicle or ABT-263, produced a 40% durable survival rate. Taken together, these observations highlight potential drug partners for the therapeutic development of Dpep.

## 1. Introduction

Abundant evidence has identified the basic leucine zipper transcription factors ATF5, CEBPB and CEBPD as having oncogenic roles in a wide variety of tumor types [1,2,3,4,5,6,7,8]. To regulate gene expression, the three factors must each form either homo- or heterodimers via their leucine zipper motifs [9,10]. To exploit this feature for potential therapeutic use, we designed a series of cell-penetrating decoy peptides that contain sequences based on the ATF5, CEBPB and CEBPD leucine zippers [8,11]. The peptides, which contain a penetratin sequence, penetrate cell membranes and act as selective dimerization competitors to suppress ATF5 and/or CEBPB and CEBPD activities. Mass spectrometry/pull-down studies using living cells indicate that one such peptide, CP-d/n-ATF5, that contains a portion of the ATF5 leucine zipper sequence, associates with and suppresses the activities of CEBPB and CEBPD [7]. Based on these findings, we designed cell-penetrating peptides designated as Bpep and Dpep that contain leucine zipper sequences of CEBPB and CEBPD, respectively, that bind and inhibit the activities of ATF5 as well as of CEBPB and CEBPD [7,8].

In vitro and in vivo studies have indicated that CP-d/n-ATF5, Bpep and Dpep pass through cell and tissue membranes and selectively promote apoptotic death of a wide range of cancer cell types while sparing normal cells [6,8,11]. Such findings support the idea that the peptides may be suitable drugs for cancer treatment [5]. While the peptides are effective as monotherapies, we have considered that they may also be used as components of combination therapies. In combination with radiation or NK cells, as well as a variety of chemotherapeutics, the peptides have consistently shown additive to synergistic activities [6,8,11,12,13]. The addition of the peptides, which appear to have few—if any—side effects in mice as monotherapies [6,8,11,12], to current treatments may therefore augment their efficacies as well as permit the use of lower levels of toxic combinatory partners. A major aim of the studies presented here is therefore to identify suitable partners that might be combined with the peptides in a clinical context.

Another consideration regarding potential clinical use of the peptides is overcoming resistance. As with many therapeutics, while the peptides effectively suppress tumor growth in vivo for prolonged periods, a portion of the tumors eventually begin to grow despite continued treatment, thus indicating development of peptide resistance [6,8,12]. In the present study, we further sought to evaluate combinations of Dpep and Bpep with agents chosen for potential synergistic activity and capacity to overcome peptide resistance.

Of relevance to the potential choice of suitable combination partners, as noted, CP-d/n-ATF5, Dpep and Bpep promote death of cancer cells by triggering apoptosis [6,8,12]. Mechanistic studies reveal that the peptides evoke both cell-context-dependent and shared changes in expression of multiple apoptosis-regulatory genes and proteins across a range of tumor cell types [6,8,12,13,14,15]. For instance, all lines tested show increases in the pro-apoptotic protein BMF as well as depletion of the survival protein survivin (product of the *BIRC5* gene) after peptide treatment [6,8,12,16]. We therefore reasoned that the combination of Dpep and Bpep with a pro-apoptotic BH3-mimetic would have the potential to show synergistic activity as well as to overcome peptide resistance. Since our studies have shown variable effects of the peptides on BCL2 and no evident effects on the related survival proteins BCLXL and BCL2L2 [6,8,12], we chose combination with ABT-263 (Navitoclax) which targets all three and which is currently in a variety of clinical trials for treatment of both solid and liquid tumors [17,18,19,20]. Our past work assessing the combination of ABT-263 with CP-dn-ATF5 indicated a degree of synergy both in several in vitro and in vivo models [6].

Of related relevance, many studies have indicated that cancer cells often undergo epigenetic changes, including hypermethylation and silencing of pro-apoptotic genes, that protects them from cell death and renders them resistant to therapeutics [21,22,23,24]. To counter this, hypomethylating agents have been developed that show clinical efficacy both as monotherapies and as combination treatments [21,22,23,24]. Such considerations suggested testing the combination of Dpep with the hypomethylating agent decitabine. Prior studies report that the combination of decitabine with the ABT-263-related BH3 mimetic Venetoclax shows clinical efficacy in treatment of AML and MDS, while the combination of decitabine with ABT-263 is in trials for these malignancies and in preclinical studies for treatment of solid tumors [25,26]. We therefore also assessed the triple combination of Dpep with ABT-263 and decitabine in the current work.

Here, we report that various combinations of Dpep with ABT-263 and decitabine synergistically suppress the growth of a variety of cultured solid and liquid tumor types and can overcome resistance to Dpep. We also show that treatment with the combination of Dpep with ABT-263 markedly enhances survival in a mouse xenograft model of melanoma.

## 2. Materials and Methods

### 2.1. Cell Culture

T98G, A375-ATCC, HCT116, MDA-MB-231 and MCF7 cells were obtained from and authenticated by the ATCC and routinely maintained in DMEM supplemented with 10% FBS and 100 U/mL Penicillin–Streptomycin at 37 °C in 5% CO_2_. These lines were examined using Universal Mycoplasma Detection Kit #30-1012K (ATCC, Manassas, VA, USA) and verified to be free from mycoplasma contamination. K562 and MOLM-14 cells were kindly provided by Drs. Azra Raza and Abdullah Ali (Columbia University, New York, NY, USA) in 2025 and were originally obtained from and authenticated by the ATCC. These lines were maintained in RPMI 1640 supplemented with 10% FBS and 100 U/mL Penicillin–Streptomycin at 37 °C in 5% CO_2_.

A375 Dpep-responsive#1 and A375 Dpep-resistant#1 cells were established as previously described [13]. A375 Dpep-responsive#2 and A375 Dpep-resistant#2 cells were generated from NCR nude mice bearing A375-ATCC melanoma xenografts that were treated 3–4 times a week with 10% glycerol in PBS or Dpep123 at a dose of 10 mg/kg via intraperitoneal injection for 8 weeks. Tumors were harvested and processed to establish cell cultures using the same tumor dissociation, cell isolation, plating, and culture procedures described above for generation of A375 Dpep-responsive#1 and Dpep-resistant#1 cells.

Dpep-resistant HCT116 cells were generated by stepwise in vitro exposure to increasing concentrations of Dpep. Briefly, HCT116 cells were initially treated with 5 μM Dpep, and the drug concentration was gradually increased (to 10, 15, and 20 μM) once the surviving cells regained a stable proliferation rate at each step. In parallel, Dpep-responsive HCT116 cells were cultured under identical conditions and passaged simultaneously but treated with 10% glycerol in PBS (pH 7.2) without Dpep. The resulting Dpep-resistant HCT116 cells were maintained in medium containing 10 µM Dpep, while the parallel Dpep-responsive HCT116 cells remained cultured in drug-free medium.

### 2.2. Peptides and Reagents

Dpep, Bpep, Dpepmut and Dpep123 peptides, subjected to TFA removal and provided as acetate salts, were purchased from Alan Scientific with the following sequences:

Dpep: RQIKIWFQNRRMKWKKLVELSAENEKLHQRVEQLTRDLAGLRQFFK

Bpep: RQIKIWFQNRRMKWKKLETQHKVLELTAENERLQKKVEQLSRELSTLRNL

FKQL

Dpep123: RQIKIWFQNRRMKWKKLVELSAENEKLHQRVEQLTR

Dpepmut: RQIKIWFQNRRMKWKKLVEGSAENEKGHQRVEQGTRDGAGGRQFFK

Peptides were dissolved in 10% glycerol in PBS (pH 7.2) and stored as 2 mM aliquots at −80 °C until further dilution for experimental use.

Decitabine (Selleckchem, Houston, TX, USA; #S1200) was purchased as a 10 mM stock solution and further diluted in DMEM for all subsequent treatments. ABT-263 (Selleckchem, #S1001) was prepared at a concentration of 20 mM by dissolving the lyophilized powder in DMSO; working solutions were then prepared by dilution in DMEM prior to use.

### 2.3. Cell Viability

T98G, A375-ATCC, A549, MCF7, K562, MOLM-14, A375 Dpep-responsive#1, A375 Dpep-resistant#1, A375 Dpep-responsive#2, A375 Dpep-resistant#2, HCT116 Dpep-responsive cells, HCT116 Dpep-resistant cells were seeded in 96-well plates, with 0.1 mL of DMEM or RPMI 1640 supplemented with 10% FBS in each well. After overnight incubation under standard culture conditions, the medium was replaced with DMEM or RPMI 1640 containing 2% FBS and the designated concentrations of Dpep, Decitabine, or ABT-263 individually or in combination and maintained for an additional 5–6 days. Cell viability of lines derived from solid tumors was determined through cell counting using either a hemocytometer or a Countess II automated cell counter (Life Technologies, Carlsbad, CA, USA). This approach avoided potential confounding effects of the peptides on cell metabolism [15] that may affect determination of cell viability by other methods.

Liquid tumor lines K562 and MOLM-14 cells were collected following drug treatment and resuspended in culture medium. An aliquot of the cell suspension was mixed 1:1 with trypan blue solution and loaded onto a hemocytometer. Viable (trypan blue–excluding) and non-viable (trypan blue–positive) cells were counted under a light microscope. This approach avoided potential confounding effects of the peptides on cell metabolism [15] that may affect determination of cell viability by other methods. Cell viability was calculated as the percentage of viable cells relative to the total cell number.

### 2.4. Tumor Cell Priming and Co-Culture with NK-92MI Cells

NK-92MI cells were obtained from the ATCC (#CRL-2408) and cultured in MyeloCult™ H5100 medium (STEMCELL Technologies, Cambridge, MA, USA; #05150) containing hydrocortisone (STEMCELL Technologies, Cambridge, MA, USA; #74142) and 12.5% FBS. Dpep-responsive HCT116 cells were seeded into 96-well plates at a volume of 0.1 mL per well in DMEM supplemented with 10% FBS. Following overnight incubation under standard culture conditions, the medium was replaced with DMEM containing 2% FBS and the indicated concentrations of Dpep, decitabine, or ABT-263, either as single agents or in combination with Dpep (i.e., Dpep + decitabine or Dpep + ABT-263). After 48 h of pre-treatment, NK-92MI cells were added at a defined effector-to-target (E:T) ratio of 2.5:1 in a volume of 0.1 mL per well. Culture medium and treatment conditions were adjusted to ensure consistent final drug concentrations and total volumes across all wells. Effector and target cells were then co-cultured for 24 h. Cell viability was assessed as previously described [13].

To evaluate whether decitabine directly affects NK-92MI cytotoxicity, Dpep-responsive HCT116 cells were pretreated with or without Dpep for 48 h, while NK-92MI cells were separately treated with or without decitabine for 24 h. The Dpep-pretreated HCT116 cells and decitabine-treated NK-92MI cells were then co-cultured for an additional 24 h, with Dpep and decitabine maintained at consistent concentrations throughout the co-culture period.

### 2.5. Plate-Seq Data Analysis

Previously reported Plate-seq data [14] derived from transcriptomic comparisons between A375 Dpep-resistant#1 and A375 Dpep-responsive#1 cells were analyzed to identify differentially expressed genes discussed in the text. Raw and processed sequencing data from the original study are publicly available in the Gene Expression Omnibus under accession number GSE244579.

### 2.6. In Vivo Antitumor Efficacy and Survival Analysis

Animal procedures were approved by the Columbia University IACUC (protocol AC-AABU1656, approved 28 December 2022) and followed guidelines set forth by the National Institutes of Health guide for care and use of laboratory animals. Female NCR nude mice (6–8 weeks old; Taconic, Rensselaer, NY, USA) were subcutaneously inoculated with 1 × 10^6^ A375-ATCC cells into the flanks. Once tumors reached a volume of 100–200 mm^3^, mice were randomized into treatment groups.

For the Dpep and ABT-263 combination study, animals were inoculated at 3 different sites on the flanks. Once tumors formed and randomization carried out, mice received intraperitoneal injections (100 µL/mouse) three times weekly of: (i) vehicle (10% glycerol in PBS), (ii) Dpep (20 mg/kg), (iii) ABT-263 (50 mg/kg), or (iv) the combination of both agents. Two independent experiments were carried out using this protocol with 5 animals in each group unless otherwise noted and the resulting data were pooled. In one experiment, one of the inoculated animals failed to form tumors and, therefore, was excluded. In this case, there were 4 animals in the control vehicle group.

For the Dpep123 study, mice were similarly inoculated at a single flank site and treated intraperitoneally with either vehicle or Dpep123 (10 mg/kg) 3–4 times weekly as indicated.

Tumor dimensions and body weights were recorded at each measurement interval. Tumor volumes (*V*) were calculated using the formula *V* = length × width^2^/2. Animal body weights were recorded at each measurement to assess systemic toxicity. For survival analysis, mice were euthanized once any single tumor reached the predefined ethical endpoint of 1000 mm^3^. In the Dpep + ABT-263 study, animals surviving to the 60-day treatment end point were maintained for another 30 days without further treatment.

### 2.7. Statistical Analyses

All individual experiments were independently conducted in triplicate, and data are presented as mean ± standard error of the mean (SEM). N values for various experiments are indicated in the figure legends. Statistical significance was determined using a two-tailed Student’s *t*-test (GraphPad Prism version 10.4.2). To assess the efficacy of Dpep123 and of Dpep, ABT-263 and DPEP + ABT-263 in suppressing tumor growth vivo, statistical significance was determined using a Mann–Whitney test in R (version 4.1.2). Survival analyses utilized Kaplan–Meier survival curves and statistical significance was determined using the log-rank test. A *p*-value < 0.05 was considered statistically significant.

Drug interactions were evaluated using the Bliss independence model as previously described [13] to determine whether the agents acted independently or exhibited synergistic effects when combined. To assess the potential synergy between Dpep, Bpep and ABT-263, drug interactions were analyzed by CompuSyn software (version 1.0) based on the Chou–Talalay median-effect method [27] using data from T98G and MCF7 cells treated with various combinations of the two agents. This approach generates normalized isobolograms in which synergy is indicated by the occurrence of points that lie to the left of the diagonal line.

## 3. Results

### 3.1. Response of Wild-Type (WT) Cells to Monotherapies

#### 3.1.1. WT Lines and Responses to Dpep

**Figure 1:**
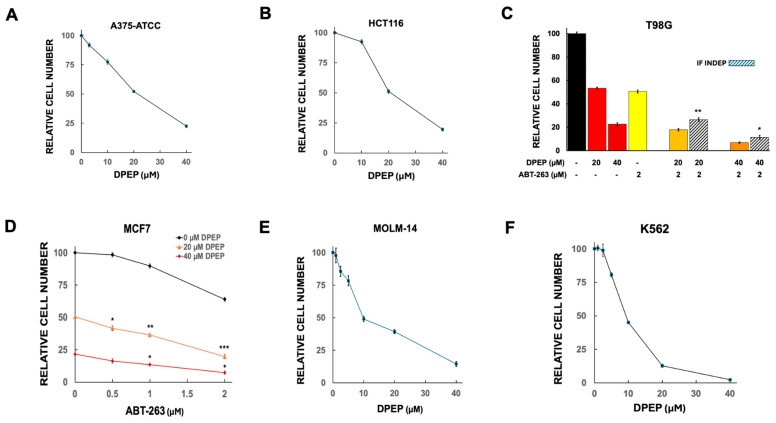
Responses of A375-ATCC, HCT116, T98G, MCF7, MOLM-14 and K562 cells to Dpep. (**A**) Dose–response relationship between Dpep concentration and cell survival for A375-ATCC cells. Cultures were exposed to Dpep at the indicated concentrations for 5–6 days and then assessed for numbers of surviving cells. Data points are given as mean ± SEM. N values for 3, 10, 20 and 40 µM Dpep are 3, 9, 21 and 12, respectively. (**B**) Dose–response relationship between Dpep concentration and cell survival for HCT116 cells. N values for 10, 20 and 40 µM Dpep are 9, 9, and 12, respectively. (**C**) Responses of T98G cells to Dpep and to ABT-263 alone and in combination. N values for 20 and 40 µM Dpep are 18 and 15, respectively. N values for ABT-263 alone and for combinations with 20 and 40 µM Dpep = 9. Cultures were exposed to the indicated treatments for 5–6 days and then assessed for numbers of surviving cells here and in subsequent panels. Values are given as mean ± SEM here and in subsequent panels. Stripped columns here and in subsequent panels show values calculated if the indicated responses to Dpep and ABT-263 were independent from one another. Here and in subsequent panels, unless otherwise indicated, asterisks indicate significance (*t*-test) of the differences between the observed values and values calculated with the assumption of independent activity. *, *p* < 0.05; **, *p* < 0.005 for comparison between observed cell number and that calculated if the various treatments were independent (here and in all following panels and figures). (**D**) Dose–response relationship between MCF7 cell number and treatments with various doses of Dpep alone and combined with the indicated concentrations of ABT-263. N for both 20 and 40 µM Dpep = 18. For 0.5, 1, and 2 µM ABT-263, N = 3, 9, and 6, respectively. For 20 and 40 µM Dpep combined with 0.5 µM ABT-263, N = 3 in both cases. For 20 and 40 µM Dpep combined with 1 µM ABT-263, N = 6 and 9, respectively. For 20 and 40 µM Dpep combined with 2 µM ABT-263, N = 6 in both cases. *, *p* < 0.05; **, *p* < 0.005; ***, *p* < 0.0005 here and in subsequent panels. (**E**) Dose–response relationship between Dpep concentration and cell survival for MOLM-14 cells. N values for 1, 2.5, 5, 10, 20, and 40 µM Dpep are 3, 3, 3, 15, 18 and 3, respectively. (**F**) Dose–response relationship between Dpep concentration and cell survival for K562 cells. N values for 1, 2.5, 5, 10, 20, and 40 µM Dpep are 3, 3, 3, 15, 12 and 3, respectively.

Prior work has established that Dpep and Bpep compromise survival of a wide range of tumor cell types, thereby broadening their potential clinical application [8,15]. In the present studies we therefore also surveyed a variety of human cancer-derived cell lines. These include lines derived from solid tumors including A375 (skin melanoma; referred here as A375-ATCC to distinguish it from sublines described below), HCT116 (colon cancer), T98G (glioblastoma) and MCF7 (breast cancer) cells and lines derived from liquid malignancies including K562 (chronic myeloid leukemia) and MOLM-14 (acute myeloid leukemia) cells. A375-ATCC, HCT116, T98G and MCF7 have previously been reported as Dpep responsive [8] and show IC50’s of about 20 µM here (Figure 1A–D). K562 and MOLM-14 cells, assessed with Dpep here for the first time, were also responsive to Dpep with IC50’s of about 9 and 10 µM, respectively (Figure 1E,F).

#### 3.1.2. WT Lines and Responses to ABT-263

**Figure 2:**
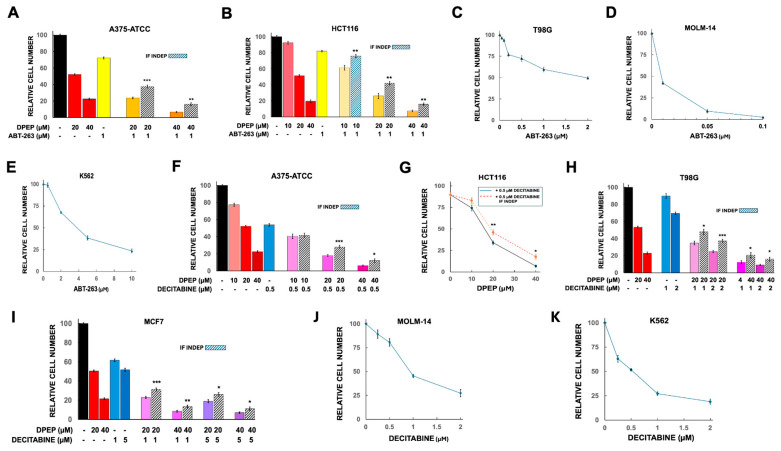
Responses of various cell lines to ABT-263 and decitabine alone and in combination with Dpep. (**A**) Responses of A375-ATCC cells to ABT-263 alone and in combinations with Dpep. Dpep values are as in Figure 1A and are shown for comparison. For ABT-263, N = 12; for ABT-263 + 20 and 40 µM Dpep, N = 6. **, *p* < 0.005; ***, *p* < 0.0005. (**B**) Responses of HCT116 cells to ABT-263 and various combinations of Dpep and ABT-263. Dpep values are as in Figure 1B and are shown for comparison. For ABT-263, N = 9; for ABT-263 + 10, 20 and 40 µM Dpep, N = 3, 3, and 6, respectively. **, *p* < 0.005. (**C**) Dose–response relationship for ABT-263 concentration and cell survival of T98G cells. For ABT-263 concentrations from 0.05 to 1 µM, N = 3, for 2 µM ABT-263, N = 9. (**D**). Dose–response relationship between ABT-263 concentration and cell survival for MOLM-14 cells. N values for 0.01, 0.05, and 0.1 µM ABT-263 are 9, 3, and 3 respectively. (**E**) Dose–response relationship between ABT-263 concentration and cell survival for K562 cells. N values for 0.5, 2, 5 and 10 µM µM ABT-263 are 3, 12, 3 and 3 respectively. (**F**) Responses of A375-ATCC cells to decitabine and to various combinations of Dpep + decitabine. Dpep values are as in Figure 1A and are shown for comparison. For decitabine, N = 15; for decitabine + 10, 20 and 40 µM Dpep, N = 6, 12, and 12, respectively. *, *p* < 0.05; ***, *p* < 0.0005. (**G**) Responses of HCT116 cells to decitabine alone and to various combinations of Dpep and 0.5 µM decitabine. N = 9, 6, 9 and 6 for 0, 10, 20 and 40 µM Dpep + 0.5 µM decitabine. Dotted red line shows values calculated if the indicated responses to Dpep and decitabine were independent from one another. *, *p* < 0.05; **, *p* < 0.005. (**H**) Responses of T98G cells to decitabine alone and to various combinations of Dpep and decitabine. Dpep values are as in Figure 1C and are shown for comparison. For 1 and 2 µM decitabine alone, N = 6 and 12, respectively. For combinations of 20 and 40 µM Dpep with 1 and 2 µM decitabine, N = 6, 12, 6 and 12, respectively. *, *p* < 0.05; ***, *p* < 0.0005. (**I**) Responses of MCF7 cells to decitabine alone and to various combinations of Dpep and decitabine. Dpep values are as in Figure 1D and are shown for comparison. For 1 and 5 µM decitabine alone, N = 12 and 9, respectively. For combinations of 20 and 40 µM Dpep with 1 and 5 µM decitabine, N = 12, 9, 12 and 9, respectively. *, *p* < 0.05; **, *p* < 0.005; ***, *p* < 0.0005. (**J**) Dose–response relationship between decitabine concentration and cell survival for MOLM-14 cells. N values for 0.25, 0.5, 1 and 2 µM decitabine are 3, 3, 15 and 3, respectively. (**K**) Dose–response relationship between decitabine concentration and cell survival for K562 cells. N values for 0.25, 0.5, 1 and 2 µM decitabine are 3, 12, 6 and 3, respectively. 3.1.3. WT Lines and Responses to Decitabine.

As shown in Figure 1C and Figure 2A–E, most of the lines assessed in our hands had IC50’s for ABT-263 of greater than 1 µM with an apparent plateau in efficacy at higher concentrations. MOLM-14 cells were the one exception with an IC50 for ABT-263 of about 8 nM (Figure 2D). This is consistent with a prior report that this line has an IC50 for the related compound venetoclax of 10 nM [28].

In our studies, A375-ATCC and K562 cells exhibited IC50’s for decitabine of about 0.5 µM and while the remaining lines were less sensitive with apparent IC50 values of 1 µM or greater and appearing in several cases to reach a plateau in activity (Figure 2F–K).

### 3.2. Response of WT Cells to Dual Therapies

We next assessed the responses of each of the WT lines to dual combinations of Dpep, ABT-263 and decitabine. We tested a range of combinations to identify those that might produce the greatest synergy as well as efficacy. We were particularly interested in identifying conditions that reduced the surviving population by greater than 90%. Where possible, we tested drug concentrations that promoted a roughly 50% (or greater) reduction in cell numbers as monotherapies. The literature indicates that serum concentrations of ABT-263 and decitabine used for clinical treatment generally reach maximal levels of about 2 and 5 µM, respectively [17,29,30]. We therefore employed these drugs at levels below these limits in our studies, even in cases in which they showed less than 50% reduction in cell numbers as monotherapies. For the various combinations, we routinely used the Bliss Independence model [31] to determine whether the drugs act independently in combination and show a degree of synergy.

#### 3.2.1. Combinations of Dpep with Decitabine

**Figure 3:**
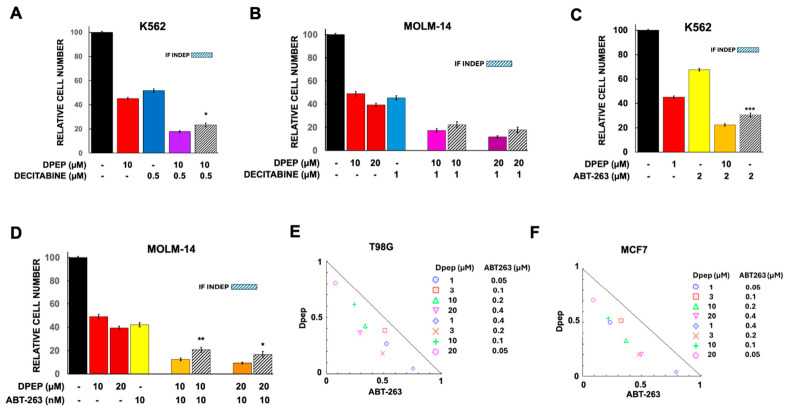
Responses of various cell lines to combinations of Dpep with ABT-263 and decitabine. (**A**) Response of K562 cells to a combination of Dpep and decitabine. Dpep and decitabine values are as in Figure 1F and Figure 2K, respectively, and are shown for comparison. For the Dpep combination with decitabine, the N values is 9. *, *p* < 0.05. (**B**) Responses of MOLM-14 cells to various combinations of Dpep and decitabine. Dpep values are as in Figure 1E and decitabine values as in Figure 2J and are shown for comparison. For 10 and 20 µM Dpep combinations with decitabine, N values are 6 and 12, respectively. (**C**) Response of K562 cells to a combination of Dpep and ABT-263. Dpep and ABT-263 values are as in Figure 1F and Figure 2E, respectively and are shown for comparison. For the Dpep and ABT-263 combination, the N value is 9. ***, *p* < 0.0005. (**D**) Responses of MOLM-14 cells to various combinations of Dpep and ABT-263. Dpep values are as in Figure 1E and ABT-263 values as in Figure 2D and are shown for comparison. For 10 and 20 µM Dpep combinations with ABT-263, N values are 6 and 9, respectively. *, *p* < 0.05; **, *p* < 0.005. (**E**) Isobologram indicating synergistic interaction between Dpep and ABT-263 in T98G cells. (**F**) Isobologram indicating synergistic interaction between Dpep and ABT-263 in MCF7 cells.

The combination of Dpep with decitabine indicated significant synergy between the two drugs in A375-ATCC, HCT116, T98G, MCF7 and K562 cells (Figure 2F–I and Figure 3A). This combination also showed a trend towards synergy in MOLM-14 cells (Figure 3B). For A375-ATCC, HCT116, T98G and MCF7 cells, elimination of >90% of cells was achieved with the combination of 10–40 µM Dpep and 0.5–2 µM decitabine (Figure 2F–I). For the K562 and MOLM-14 lines, >80% of cells were eliminated after exposure to 10 µM Dpep + 0.5 µM decitabine and 10 or 20 µM Dpep + 1 µM decitabine, respectively (Figure 3A,B).

#### 3.2.2. Combination of Dpep + ABT-263

Dpep combined with ABT-263 yielded significant synergistic activity in all lines assessed and in a variety of combinatory ratios (Figure 1C,D, Figure 2A,B and Figure 3C,D). To assess synergy, we also carried out CompuSyn analysis on T98G and MCF7 cells treated with various combinations of Dpep and ABT-263. Isobolograms based on the analyses supported synergistic activity (Figure 3E,F). In 4/6 lines, elimination of >90% of the cells was achieved with the combination of 20–40 µM Dpep and 1–2 µM ABT-263 (Figure 1C,D and Figure 2A,B). For MOLM-14 cells, which are particularly sensitive to ABT-263, this level of elimination occurred with 20 µM Dpep combined with 10 nM ABT-263 (Figure 3D). For K562 cells, which are relatively insensitive to ABT-263, elimination of 80% of cells was observed with 10 µM Dpep + 2 µM ABT-263 (Figure 3C).

#### 3.2.3. Required Cotreatment Time with Dpep and ABT-263

**Figure 4:**
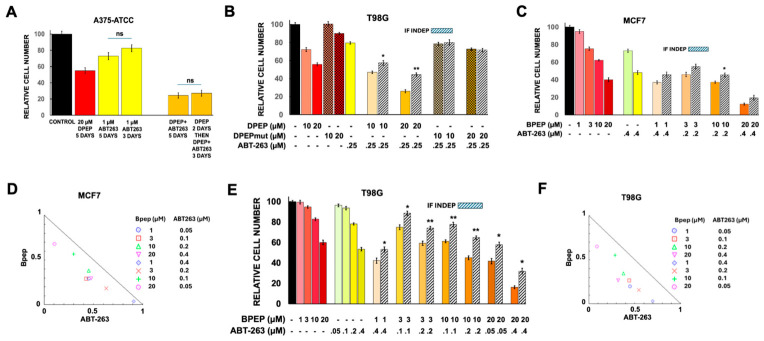
Responses of tumor cell lines to combinations of ABT-263 with Dpep, a Dpep mutant or Bpep. (**A**) Effect of co-treatment time on outcome of Dpep plus ABT-263 efficacy. A375-ATCC cells were exposed to either 2 days of treatment with 20 µM Dpep followed by 3 days’ exposure to 20 µM Dpep + 1 µM ABT-263 or to the full combination for 5 days and in each case evaluated for relative numbers of surviving cells. N = 3 for all conditions. ns = not significant. (**B**) Dpep that is mutated to disrupt its leucine zipper (Dpepmut) does not synergize with ABT-263. T98G cells were treated with the indicated agents and assessed for survival. N values for all conditions = 3. *, *p* < 0.05; **, *p* < 0.005. (**C**) The combination of Bpep with ABT-263 indicates synergistic action on T98G cells. N values for 1 µM Bpep are 3 and for all other concentrations, 6. For all other treatments, N = 3. *, *p* < 0.05. (**D**) Isobologram indicates synergistic interaction between Bpep and ABT-263 in MCF7 cells. (**E**) The combination of Bpep with ABT-263 indicates synergistic action on T98G cells. N values for 1 µM Dpep are 3 and for all other concentrations, 6. For all other treatments, N = 3. *, *p* < 0.05; **, *p* < 0.005. (**F**) Isobologram indicates synergistic interaction between Bpep and ABT-263 in T98G cells.

In prior studies, we observed that optimal killing with Dpep occurs by 5–6 days of treatment [8] and, therefore, used this time frame for our monotherapy and combination experiments. To determine whether the entire time is required for the combination of Dpep with ABT-263, we compared the effects on A375-ATCC cells of 5 days treatment with the full combination with those achieved by 2 days of Dpep pretreatment alone, followed by 3 days exposure to the full combination. The outcome (Figure 4A) indicated that the two treatment protocols produced similar efficacy.

#### 3.2.4. The Dpep Leucine Zipper Domain Is Required for Full Activity and Synergy with ABT-263

Dpep is designed to interfere with ATF5, CEBPB and CEBPD activity by means of specific interactions with their leucine zippers. Past work has established that a mutated form of Dpep (Dpepmut) in which the key leucines in the zipper have been replaced with glycine residues shows greatly reduced capacity to promote cancer cell death [8]. Here, we assessed the ability of Dpepmut to show synergistic activity with ABT-263. T98G cells were treated with combinations of ABT-263 with either Dpep or Dpepmut. As shown in Figure 4B, in contrast to Dpep, Dpepmut showed little effect on cell number alone and no synergy with ABT-263. These observations indicate that the synergistic actions of Dpep with ABT-263 are not due to off-target effects or non-specific actions of the peptide and are mediated via leucine zipper interactions.

#### 3.2.5. Bpep Shows Synergy with ABT-263

Although Bpep has a very different leucine zipper sequence than Dpep, it too targets ATF5, CEBPB and CEBPD and, at similar concentrations, selectively compromises cancer cell survival and growth [8]. We tested the combination of Bpep with ABT-263 on T98G and MCF7 cells and noted evidence for synergistic effects similar to those seen with Dpep (Figure 4C–F).

#### 3.2.6. Combinations of ABT-263 with Decitabine

**Figure 5:**
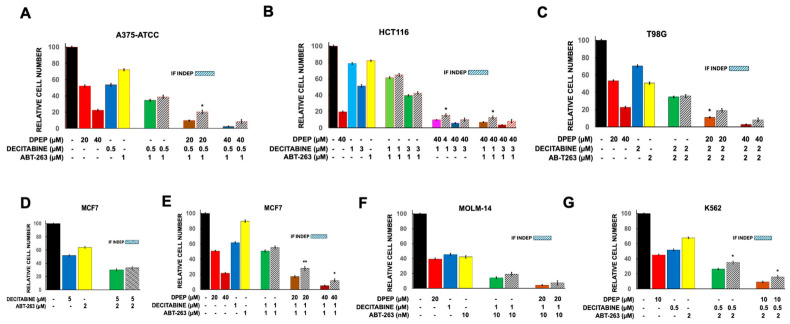
Responses of various tumor cell lines to combinations of Dpep, ABT-263 and decitabine. (**A**) Responses of A375-ATCC cells to various combinations of Dpep, ABT-263 and decitabine. Values for Dpep, ABT-263 and decitabine treatments alone are as in Figure 1A and Figure 2A,F, respectively and are shown for comparison. For ABT-263 + decitabine, N = 6; for the combinations of 20 µM and 40 µM Dpep + decitabine and ABT-263, N = 6 in both cases. *, *p* < 0.05. (**B**) Responses of HCT116 cells to various combinations of Dpep, ABT-263 and decitabine. Dpep, ABT-263 and decitabine values are as in Figure 1B and Figure 2B,G respectively and are included for comparison. For 1 and 3 µM decitabine and all other combinations, N = 6. *, *p* < 0.05. (**C**) Responses of T98G cells to various combinations of Dpep, ABT-263 and decitabine. Values for Dpep, ABT-263 and decitabine are as given in Figure 1C and Figure 2C,H, respectively and are included for comparison. For decitabine + ABT-263, N = 6. For 20 and 40 µM Dpep in combination with ABT-263 and decitabine, N = 6 in both cases. *, *p* < 0.05. (**D**) The combination of ABT-263 (2 µM) and decitabine (5 µM) does not show synergy on MCF7 cells. Values for ABT-263 and decitabine treatments are as given in Figure 1D and Figure 2I, respectively and are included for comparison. For the ABT-263 + decitabine combination, N = 3. (**E**) Responses of MCF7 cells to various combinations of Dpep, ABT-263 and decitabine. Values for Dpep, ABT-263 and decitabine alone are as given in Figure 1D and Figure 2I and are included for comparison. For decitabine + ABT-263, N = 6. For 20 and 40 µM Dpep in combination with ABT-263 and decitabine, N = 6 in both cases. *, *p* < 0.05; **, *p* < 0.005. (**F**) Responses of MOLM-14 cells to various combinations of Dpep, ABT-263 and decitabine. Values for Dpep, ABT-263 and decitabine are as given in Figure 1E and Figure 2D,J, respectively and are included for comparison. For decitabine + ABT-263, N = 6. For 20 µM Dpep in combination with ABT-263 and decitabine, N = 6. (**G**) Response of K562 cells to a combination of Dpep, ABT-263 and decitabine. Values for Dpep, ABT-263 and decitabine alone are as given in Figure 1F and Figure 2E,K, respectively, and are included for comparison. For decitabine + ABT-263, N = 6. For Dpep in combination with ABT-263 and decitabine, N = 6. *, *p* < 0.05.

In the course of our studies, we also assessed the combination of ABT-263 with decitabine (Figure 5). With the exception of K562 cells, the combination of ABT-263 and decitabine did not indicate significant synergistic activity in the tested lines (Figure 5A–G).

### 3.3. Responses of WT Cells to the Triple Combination of Dpep + ABT-263 + Decitabine

As with the dual combinations, the triple treatment with Dpep, ABT-263 and decitabine showed context-dependent combinatory actions depending on cell line. In all lines tested, appropriate sets of concentrations were identified that brought about the loss of >90% of the cells (Figure 5A–C,E–G). This included Dpep concentrations from 10–40 µM, 0.5–3 µM decitabine and 0.01 to 1–2 µM ABT-263. For 5/6 lines assessed, at least one triple combination showed significant synergy, with the 6th line (MOLM-14 cells) showing a trend towards significance (Figure 5F). It should be noted that in experiments with >90% cell loss, the numbers of remaining cells were in many cases too low to achieve statistical significance.

### 3.4. Dpep-Resistant Cells

Like many cancer drugs, while Dpep suppresses growth of tumor cells in mouse models and prolongs survival of tumor-bearing mice, resistant tumors eventually emerge [8]. Given the efficacy of combinations of Dpep with ABT-263 and decitabine on wild-type (WT) cancer lines, we asked whether such combinations would also be effective on cells that develop resistance to Dpep.

#### 3.4.1. Generation and Characterization of Dpep-Resistant HCT116 Cells and Their Responses to Combinations of Dpep, ABT-263 and Decitabine

**Figure 6:**
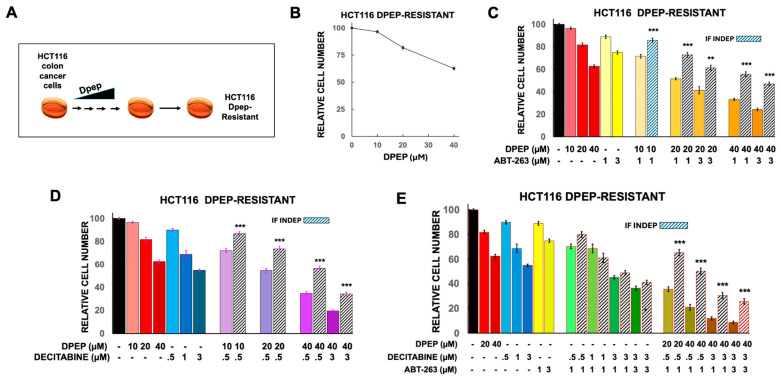
Generation of Dpep-resistant HCT116 cells and their responses to combinations comprising Dpep, ABT-263, and decitabine. (**A**) Scheme for selection of HCT116 cells resistant to Dpep. (**B**) Dose–response relationship between Dpep concentration and cell survival for HCT116 Dpep-resistant cells. N values for 10, 20, and 40 µM Dpep are 24, 24 and 27, respectively. (**C**) Response of HCT116 Dpep-resistant cells to ABT-263 and to combinations of Dpep and ABT-263. Dpep values are as in (**B**) and are included for comparison. For ABT-263 at 1 and 3 µM, the N values are 18 and 9, respectively. For the combinations of 10 µM Dpep, 1 µM ABT-263; 20 µM Dpep, 1 and 3 µM ABT-263; and 40 µM Dpep, 1 and 3 µM ABT-263, the N values are 9, 9, 3, 12 and 6, respectively. **, *p* < 0.005; ***, *p* < 0.0005. (**D**) Response of HCT116 Dpep-resistant cells to decitabine and to combinations of Dpep and decitabine. Dpep values are as in (**B**) and are included for comparison. For decitabine at 0.5, 1 and 3 µM, the N values are 15, 6 and 12, respectively. For the combinations of 10 µM Dpep, 0.5 µM decitabine; 20 µM Dpep, 0.5 µM decitabine; and 40 µM Dpep, 0.5 and 3 µM decitabine, the N values are 6, 12, 6 and 12, respectively. ***, *p* < 0.0005. (**E**) Response of HCT116 Dpep-resistant cells to combinations of Dpep, ABT-263 and decitabine. Values for Dpep, ABT-263 and decitabine alone are as given in previous panels and are included for comparison. For decitabine + ABT-263 combinations of 0.5 µM + 1 µM, 1 µM + 1 µM, 3 µM + 1 µM and 3 µM + 3 µM, N = 3, 6, 6 and 6, respectively. For Dpep + decitabine + ABT-263 combinations of 20 µM + 0.5 µM + 1 µM; 40 µM + 0.5 µM + 1 µM; 40 µM + 3 µM + 1 µM; and 40 µM + 3 µM + 3 µM, N = 3, 3, 6 and 6, respectively. ***, *p* < 0.0005.

As one approach to obtaining cells resistant to Dpep, we carried out an in vitro selection in which HCT116 cells were cultured over time with increasing concentrations of the peptide (Figure 6A). Untreated cultures were maintained in parallel for the same period of time and served as a control. In contrast to the control cells with an IC50 of about 20 µM for Dpep (Figure 1D), the Dpep-resistant HCT116 cells had an IC50 of >40 µM (Figure 6B). Despite their blunted response to Dpep, the resistant cells maintained sensitivities to decitabine and ABT-263 that were similar to those of control cells (Figure 6C,D). Also, as with control cells, the decitabine + ABT-263 combination failed to show synergy in the resistant cells (Figure 6E). In contrast, the resistant cells exhibited strong, significant synergistic responses to Dpep in combination with either or both decitabine and ABT-263 (Figure 6C–E). Moreover, we were able to identify triple combinations of Dpep, decitabine and ABT-263 that reduced the numbers of the resistant cells by >90% (Figure 6E).

#### 3.4.2. Generation of Dpep-Resistant and Dpep-Responsive A375 Cells from Tumors

**Figure 7:**
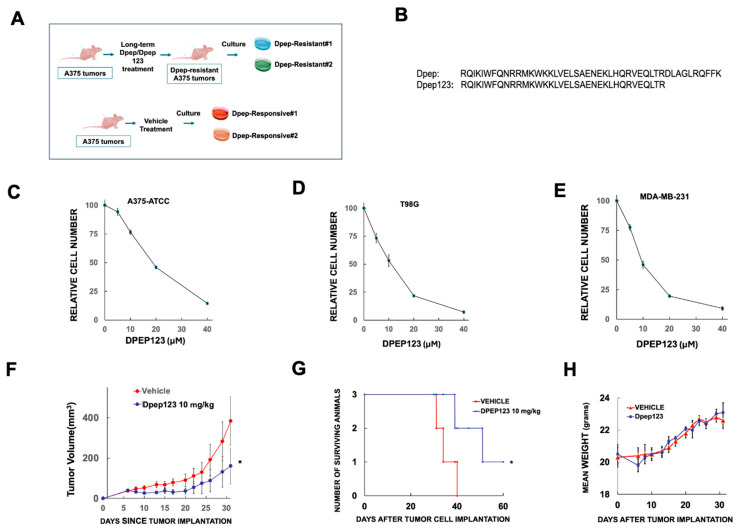
Generation of Dpep-resistant A375 cell lines and characterization of Dpep123. (**A**) Scheme for generation of Dpep-resistant lines by culturing xenografts of A375-ATCC cells that showed in vivo resistance to Dpep or Dpep123. (**B**) Comparison of the amino acid sequences of Dpep and Dpep123. (**C**–**E**) Dose–response relationship between Dpep123 concentration and cell survival for (**C**), A375-ATCC; (**D**), T98G; and (**E**), MDA-MB-231 breast cancer cells. For all lines, N = 6. (**F**) Growth curves of A375-ATCC subcutaneous xenografts in mice treated either with vehicle or 10 mg/kg Dpep123. * *p* = 0.042, Dpep123 treated vs. Vehicle (Wilcoxon Rank-Sum test). N = 3 for each treatment condition. (**G**) Animal (bearing tumors as in (**E**)) survival vs. time for treatments with either vehicle or 10 mg/kg Dpep123. N = 3 animals/group. * *p* = 0.0495 for vehicle vs. Dpep123. (**H**) Animal (bearing tumors as in (**E**)) weight as a function of time of treatment with either vehicle of 10 mg/kg Dpep123.

As an additional strategy to obtain Dpep-resistant cells, we generated mouse subcutaneous A375-ATCC melanoma xenografts and treated them with either Dpep or an active, shortened (by 10 amino acids at the C terminus) form of Dpep referred to as Dpep123 (Figure 7A,B). Dpep123 exhibits in vitro and in vivo anti-tumor activities similar to those of Dpep (Figure 7B–H). As seen previously with Dpep treatment [8], tumor growth was initially suppressed for several weeks, but after prolonged treatment, tumors eventually reappeared. One such tumor from a Dpep-treated animal and one from an animal treated with Dpep123 were harvested, dissociated and used to establish polyclonal cultures designated as A375 Dpep-resistant#1 and A375 Dpep-resistant#2, respectively. In addition, we also harvested two comparably sized tumors from two different mice inoculated with A375-ATCC cells, but treated with vehicle, and used these to establish control cultures designated as A375 Dpep-responsive#1 and A375 Dpep-responsive#2, respectively (Figure 7A).

#### 3.4.3. Response of Dpep-Resistant and Dpep-Responsive A375 Cells to Mono- and Combination Therapies

**Figure 8:**
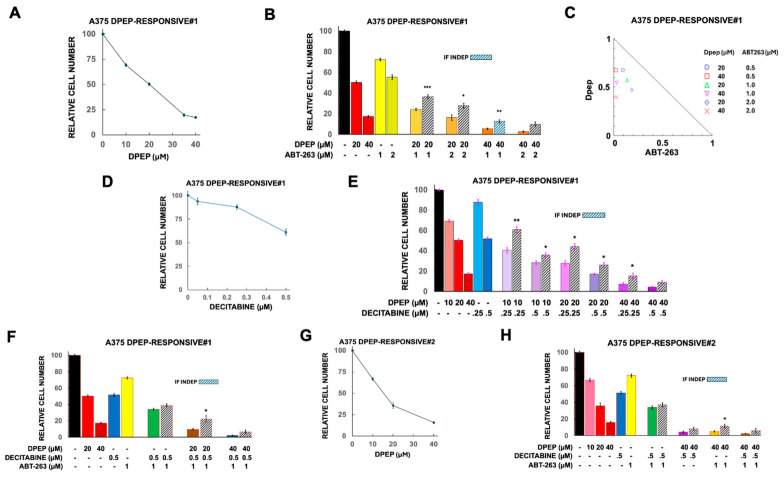
Responses of A375 Dpep-responsive cells to combinations comprising Dpep, ABT-263, and decitabine. (**A**) Dose–response relationship between Dpep concentration and cell survival for A375 Dpep-responsive#1 cells. N values for 10, 20, 35 and 40 µM Dpep are 12, 18, 6 and 15, respectively. (**B**) Response of A375 Dpep-responsive#1 cells to ABT-263 and to combinations of Dpep and ABT-263. Dpep values are as in (**B**) and are included for comparison. For ABT-263 at 1 and 2 µM, the N values are 9 and 3, respectively. For the combinations of 20 µM Dpep with 1 and 2 µM ABT-263, N values are 9 and 3, respectively. For the combinations of 40 µM Dpep with 1 and 2 µM ABT-263, N values are 9 and 3, respectively. *, *p* < 0.05; **, *p* < 0.005; ***, *p* < 0.0005. (**C**) Isobologram indicating synergistic interaction between Dpep and ABT-263 in A375 Dpep-responsive#1 cells. (**D**) Dose–response relationship between decitabine concentration and cell survival for A375 Dpep-responsive#1 cells. N values for 0.05, 0.25, and 0.5 µM decitabine are 6, 6 and 12, respectively. (**E**) Response of A375 Dpep-responsive #1 cells to combinations of Dpep and decitabine. Dpep values are as in (**B**) and are included for comparison. For the combinations of 10, 20 and 40 µM Dpep, with 0.25 and 0.5 µM decitabine, N values are 3, 6, 3, 9, 3 and 9, respectively. *, *p* < 0.05; **, *p* < 0.005. (**F**) Response of A375 Dpep-responsive #1 cells to combinations of Dpep, ABT-263 and decitabine. Values for Dpep, ABT-263 and decitabine alone are as given in previous panels and are included for comparison. For the decitabine + ABT-263 combination, N = 6. For the combinations of 20 and 40 µM Dpep with decitabine and ABT-263, N values are 6 in each case. *, *p* < 0.05. (**G**) Dose–response relationship between Dpep concentration and cell survival for A375 Dpep-responsive#2 cells. N values for 10, 20, and 40 µM Dpep are 3, 3 and 6 respectively. (**H**) Response of A375 Dpep-responsive#2 cells to ABT-263 and decitabine and to combinations of Dpep, ABT-263 and decitabine. Values for Dpep are as given in H and are included for comparison. For the decitabine and ABT-263 alone, N = 6 in each case; for the combination, N = 6. For Dpep in combination with either decitabine or ABT-263 or combination with both, N = 6 in all cases. *, *p* < 0.05.

As shown in Figure 8A–H, the control lines generated from untreated tumors showed responses to various treatments that were quite similar to one another as well as to A375-ATCC cells. That is, they had similar sensitivities to Dpep, decitabine, and ABT-263 alone as well as to various combinations of the 3 drugs. For each Dpep-responsive line, as with A375-ATCC cells, combinations were found that diminished cell numbers by more than 90% (Figure 8B,E,F,H) and there was generally significant synergy between combinations of Dpep with ABT-263 and/or decitabine (Figure 8B,C,E,F,H).

**Figure 9:**
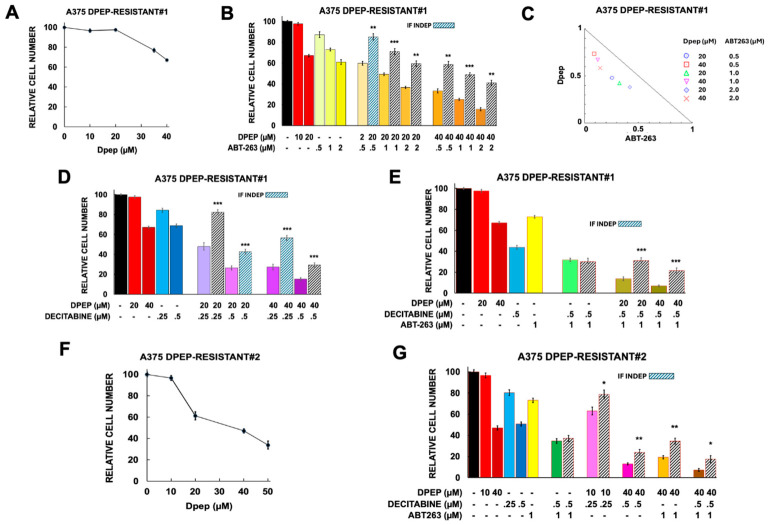
Responses of A375 Dpep-resistant#1 and A375 Dpep-resistant#2 cells to various combinations comprising Dpep, ABT-263, and decitabine. (**A**) Dose–response relationship between Dpep concentration and cell survival for A375 Dpep-resistant#1 cells. N values for 10, 20, 35, and 40 µM Dpep are 15,18, 6 and 15, respectively. (**B**) Response of A375 Dpep-resistant#1 cells to ABT-263 and to combinations of Dpep and ABT-263. **, *p* < 0.005; ***, *p* < 0.0005. Dpep values are as in (**A**) and are included for comparison. For ABT-263 at 0.5, 1 and 2 µM, the N values are 3, 9 and 3, respectively. For the combinations of 20 µM Dpep with 0.5, 1 and 2 µM ABT-263, N values are 3, 9, and 3, respectively. For the combinations of 40 µM Dpep with 0.5, 1 and 2 µM ABT-263, N values are 3, 9, and 3, respectively. (**C**) Isobologram indicating synergistic interaction between Dpep and ABT-263 in A375 Dpep-resistant#1 cells. (**D**) Response of A375 Dpep-resistant#1 cells to decitabine and to combinations of Dpep and decitabine. Dpep values are as in (**A**) and are included for comparison. For decitabine at 0.25 and 0.5 µM, the N values are 6 and 9, respectively. For the combinations of 20 µM Dpep with 0.25 and 0.5 decitabine N values are 3 and 9, respectively. For the combinations of 40 µM Dpep with 0.25 and 0.5 decitabine N values are 3 and 9, respectively. ***, *p* < 0.0005. (**E**) Response of A375 Dpep-resistant#1 cells to combinations of Dpep, ABT-263 and decitabine. Values for Dpep, ABT-263 and decitabine alone, are as in the above panels and are included for comparison. For the decitabine and ABT-263 combination, N = 6. For 20 and 40 µM Dpep in combination with decitabine and ABT-263, N = 9 in both cases. ***, *p* < 0.0005. (**F**) Dose–response relationship between Dpep concentration and cell survival for A375 Dpep-resistant#2 cells. N values for 10, 20, 40, and 50 µM Dpep are 6, 3, 6 and 3, respectively. (**G**) Responses of A375 Dpep-resistant#2 cells to decitabine and ABT-263 alone or in combination and to combinations of Dpep with decitabine or ABT-263 or with all three together. Values for Dpep are as in (**F**) and are included for comparison. N values for 0.25 and 0.5 µM decitabine are 3 and 6, respectively; for ABT-263, 6; for decitabine + ABT-263, 6; for 10 and 40 µM Dpep + decitabine, 3 and 6, respectively; for Dpep + ABT-263 and Dpep + decitabine + ABT-263, 6 in all cases. *, *p* < 0.05; **, *p* < 0.005.

Both A375 Dpep-resistant lines proved to be less sensitive to Dpep than control cells with apparent IC50 values of about 40 µM or higher (Figure 9). Sensitivities to decitabine and ABT-263, in contrast, appeared similar to those of A375-ATCC and A375 Dpep-responsive cells. Additionally, similarly to these and other lines, there was no synergy between ABT-263 and decitabine (Figure 9E,G). The two resistant lines also showed synergistic responses to combinations of Dpep with ABT-263 and/or decitabine (Figure 9B–E,G). As a result, it was possible to identify combinations of the three drugs to reduce survival in the two Dpep-resistant lines by >90% (Figure 9E,G). Taken together, these findings indicate that combinations of Dpep with decitabine and ABT-263 can effectively overcome resistance to Dpep.

#### 3.4.4. Transcriptome Comparison of Dpep-Responsive and -Resistant Cells Indicates Possible Sources of Resistance, and Rationale for Combination Therapies

We analyzed previously reported plate-seq data obtained by comparing the transcriptomes of A375 Dpep-resistant#1 cells and A375 Dpep-responsive#1 cells, both with and without 48 h of exposure to 20 µM Dpep [14]. Several key points emerged. First, resistance was not due to universal loss of Dpep sensitivity. Of 37 genes that responded to Dpep in both A375-ATCC and A375 Dpep-responsive#1 cells with logFC values of −0.95≤ or ≥0.95 (FDR < 0.05 here and in all other comparisons), 21 showed broadly similar responses in A375 Dpep-resistant#1 cells (Supplementary File S1, Sheet S1). Second, there were a number of differentially expressed genes (DEGs; LogFC −1≤ or ≥1) in the resistant cells compared with responsive cells (Supplementary File S1, Sheet S2). Among these, over 200 (of which about 70% were down-regulated in resistant cells) were detected without Dpep treatment and did not significantly respond to Dpep in either line. An additional 26 DEGs (20 of which were down-regulated in Dpep-resistant cells) were found before Dpep treatment and responded to Dpep only in Dpep-responsive cells so as to further enhance differential expression between the two lines (Supplementary File S1, Sheet S3). Third, among up-regulated DEGs in the resistant line, eight were identified in the literature as oncogenes (Supplementary File S1, Sheets S4 and S5) while 14 that were down-regulated have been described as tumor suppressors (Supplementary File S1, Sheets S6 and S7). In several cases, such roles have been reported in melanoma cells (Supplementary File S1, Sheets S4–S7). Fourth, a number of the up-regulated oncogenes inhibit apoptosis while several of the down-regulated tumor suppressors promote apoptosis (Supplementary File S1, Sheets S4–S7). Fifth, many of the down-regulated tumor suppressors are reported as hypermethylated in cancer cells and subject to upregulation by decitabine (Supplementary File S1, Sheets S6 and S7). Taken together, these observations support the hypotheses that resistance to Dpep includes upregulation of oncogenic genes and epigenetic downregulation of tumor suppressor genes that act to favor cell survival. Such considerations provide both a rationale for combining Dpep with ABT-263 and decitabine and a potential mechanism behind the observed effects of such combinations.

### 3.5. Pretreatment of Tumor Cells with Dpep Combined with Either Decitabine or ABT-263 Synergistically Sensitizes Them to Killing by NK-92MI Cells

Natural killer (NK) cells play an important role in the innate response to malignant cells by promoting their apoptotic death. We recently observed that a two-day pretreatment with Dpep sensitizes a variety of tumor cell types to the killing actions of the NK cell line NK-92MI [13]. Such findings support the possibility that Dpep may enhance the efficacy of endogenous and/or exogenously supplied NK cells in attacking tumors. Given our findings here that ABT-263 and decitabine synergize with Dpep, we asked whether their inclusion during tumor cell pre-treatment with Dpep would elicit synergistic sensitization to NK-92MI cell killing. Accordingly, we pre-treated HCT116 cells for 48 h with 20 µM Dpep alone or in combination with either 1 µM ABT-263 or 1 µM decitabine and then for an additional 24 h exposed them to the same treatments plus NK-92MI cells followed by determination of surviving cell numbers (Supplementary Figure S1A,C). At the doses employed, Dpep does not affect NK-92MI cell numbers [13] and neither ABT-263 nor decitabine had an appreciable effect on NK-92MI cell survival (NK-92MI cell survival proportions after 24 h exposure to 1 µM decitabine or ABT-263 were 92 ± 2% (N = 6) and 100 ± 4 (N = 3), respectively). ABT-263 and decitabine pre-treatment alone each sensitized HCT116 cells to NK-92MI killing and to an extent similar to that promoted by Dpep alone (Supplementary Figure S1B,D). The pre-treatment combinations of Dpep with either ABT-263 or decitabine yielded an even greater degree of sensitization compared to that anticipated if each agent acted independently (Supplementary Figure S1B,D). Because decitabine may have direct actions on NK-92MI cells that affect their cytotoxic activity, we compared the effects of naive NK-92MI cells on HCT116 cells with those of NK-92MI cells pre-treated with decitabine for 24 h and then co-cultured with HCT116 cells for another 24 h in the continued presence of decitabine (Supplementary Figure S1E). A similar experiment was carried, but with HCT116 cells pre-treated with Dpep alone (Supplementary Figure S1E). In both cases, pre-treating NK-92Mi cells with decitabine had no significant effect on the outcome (Supplementary Figure S1F), indicating that its sensitizing actions are on the HCT116 rather than on NK-92MI cells.

### 3.6. Efficacy of the Dpep-ABT-263 Combination In Vivo

**Figure 10:**
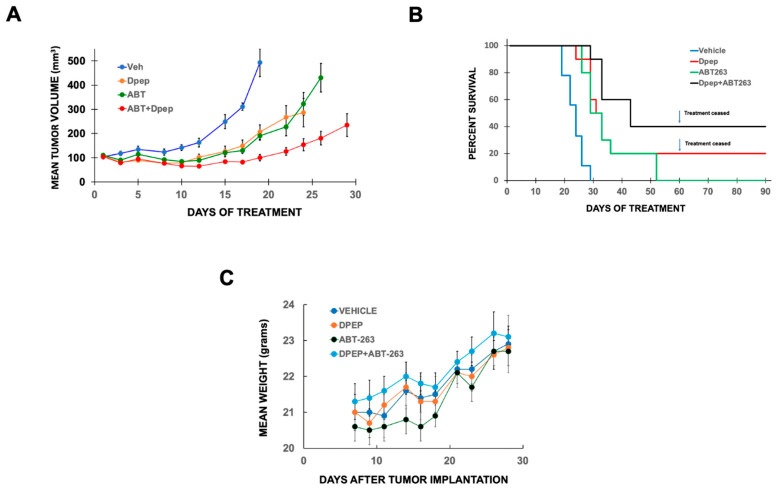
In vivo efficacy of a Dpep + ABT-263 combination a xenograft model of melanoma. (**A**) Growth curves of A375-ATCC subcutaneous xenografts in mice treated either with vehicle, 50 mg/kg ABT-263, 20 mg/kg Dpep or 50 mg/kg ABT-263 + 20 mg/kg Dpep. N = 9 for vehicle and 10 for all other treatments. *p* values for the combination vs vehicle, 0.0011; for the combination vs. ABT-263, 0.0388; for the combination vs. Dpep 0.006. (**B**) Animal (bearing tumors as in (**A**)) survival vs, time for treatments as in (**A**). *p* values are given in the text. (**C**) Mean animal (bearing tumors as in (**A**)) weights ± SEM over time for treatments as in (**A**). N = 9–10 per condition.

To further assess the efficacy of drug combinations with Dpep, we carried out an in vivo study of subcutaneous A375-ATCC mouse xenografts (generally 3 per subject). When tumors became palpable, the animals were treated 3x/week until day 60 with either vehicle, 20 µg/kg Dpep, 50 mg/kg ABT-263 or the combination of Dpep + ABT-263. As shown in Figure 10A, treatments with Dpep or ABT-263 alone each significantly slowed tumor growth to a similar extent. Both treatments also prolonged survival (*p* = 0.0021 for Dpep vs. vehicle; 0.005 for ABT-263 vs. vehicle) and in the case of Dpep treatment alone, 2/10 mice survived to the pre-determined 60-day endpoint (Figure 10B). For the Dpep + ABT-263 combination, there was a substantially more effective suppression of tumor growth compared with that promoted by the individual treatments (Figure 10A). The combination also promoted a significantly enhanced survival time (*p* = 0.0021 for the combination vs. vehicle; *p* = 0.0214 for the combination vs ABT-263; and *p* = 0.0431 for the combination vs. Dpep; log rank test) (Figure 10B). In this case, 4/10 mice survived to the 60-day treatment endpoint. For all mice surviving at 60 days, no palpable tumors were detected. Treatments were then ceased and the mice were observed for another 30 days. In each case, tumors were not detected by palpation or histology and appeared to be eradicated. As in past work, the single and combined treatments did not affect mean animal weight (Figure 10C) or appear to cause evident side effects [6,8].

## 4. Discussion

Past studies indicate that cell-penetrating peptides that target ATF5, CEBPB and CEBPD have the potential to act as treatments for a wide range of tumor types and to do so with a high degree of safety [5,6,8,11,12]. Here, our aim has been to identify and assess drugs currently in clinical use or trials, that can be combined with our peptides to enhance efficacy and to overcome peptide resistance. We present findings for two such drugs ABT-263, a BCL2 family inhibitor, and decitabine, a hypomethylating agent. In doing so, we surveyed a range of different tumor cell types as well as lines selected for resistance to our peptides.

Our data consistently show that Dpep combines well with ABT-263 and decitabine, either as dual combinations or in combinations of all three. In our studies, we surveyed six cell lines derived from tumors of diverse origins for their vulnerability to Dpep alone or in combinations with ABT-263 and decitabine. All responded to Dpep with IC50’s of 10–20 µM. All also showed significant synergistic responses to the combination of Dpep and ABT-263 and all but MOLM-14 cells showed significant synergistic responses to combinations of Dpep with decitabine or of Dpep with ABT-263 + decitabine. These observations suggest that the combination of Dpep with a BH3 mimetic and a hypomethylating agent may have synergistic efficacy across a broad range of tumor types.

Among our goals was to find combinations of the drugs that would result in at least a 90% decrease in numbers of cultured tumor cells, including lines selected for Dpep resistance. Although each tumor line showed context-dependent responses to the individual agents and combinations thereof, we were able to identify combination treatments in each case that achieved this goal. Moreover, for the combinations employed, we used levels of ABT-263 and decitabine that appear to be compatible with those safely used in human studies. It should be noted that while we sought combinations that reduced tumor cell numbers by greater than 90%, in many cases this was surpassed, and even further reduction was likely possible within the concentration limits that we considered.

Our past work has principally focused on the actions of our peptides on solid tumors. Here, we expanded our scope to include liquid tumor lines K562 (CML) and MOLM-14 (AML). Both proved to be sensitive to Dpep monotherapy (IC50 9–10 µM). Combinations of Dpep with ABT-263 and/or decitabine were also found that synergized to reduced cell numbers of these lines by >90%. BCL2 family inhibitors and hypomethylating agents are presently employed as both monotherapies and in combination for treatment of various liquid tumors [19,25,32,33]. However, successful treatment is limited by potential side effects and development of resistance. Our findings suggest that Dpep may be useful as an alternative treatment for leukemias as a monotherapy or may be used as part of a combination to enhance overall efficacy and to overcome resistance. The apparent safety of Dpep and its synergy with ABT-263 and decitabine may also promote the use of such agents in combination therapies at reduced doses in both liquid and solid tumors, and thus, decreasing the likelihood of adverse side effects.

As an initial approach to evaluating the efficacy of Dpep combinations in vivo, we tested the Dpep + ABT-263 combination in a mouse subcutaneous melanoma xenograft model. While each monotherapy reduced the rate of tumor growth and prolonged survival, the combination was significantly more effective by both measures. Moreover, the combination produced a durable 40% survival rate (compared with no survival for vehicle or ABT-263 treated animals). Clearly, additional in vivo studies of this type are warranted.

Given that the major aim of this study has been to identify drugs in clinical use or in trials with which Dpep may be combined to achieve synergistic death of tumor cells and to overcome Dpep resistance, we have not focused here on the molecular mechanisms that underlie these effects. Nevertheless, our past findings [8] that our peptides promote apoptotic tumor cell death via upregulation of pro-apoptotic proteins (such as BMF) and down-regulation of survival proteins (such as survivin) raises the hypothesis that ABT-263, which inhibits pro-survival BCL2, BCLXL and BCL-W [20], combines with Dpep to drive cells beyond an apoptotic threshold. Similarly, decitabine, which appears to modulate the epigenome to promote apoptosis [23], may also combine with Dpep to push cells beyond their capacity for survival. In any case, further experimental exploration is needed to clarify the bases for the synergism of our peptides with ABT-263 and decitabine. Similar hypotheses may pertain to the mechanisms by which Dpep sensitizes HCT116 cells to NK-92MI cell killing and by which this is augmented by combination with ABT-263 and decitabine [13].

As a part of our study, we carried out transcriptional profiling to gain insight regarding the potential mechanisms of Dpep resistance and how this might be overcome. A past Plate-seq study that included various cancer cell types indicated that Dpep triggers a set of cell-context-dependent transcriptional changes that favor apoptotic cell death [14]. The present analysis indicates that resistance was not due to global loss of Dpep responsiveness. Rather, resistant cells showed selective loss of a subset of Dpep responses as well as up-regulation of a number of oncogenes and down-regulation of a suite of tumor repressors. We noted that many of the up-regulated oncogenes have reported anti-apoptotic actions while a number of the down-regulated tumor suppressors can otherwise trigger pro-apoptotic actions. Such observations provide a potential rationale and mechanistic explanation for the synergistic effects of combining Dpep with a BCL2 family inhibitor, both in WT and Dpep-resistant cells.

Hypermethylation and consequent repression of genes associated with regulation of growth and survival represents a contributary mechanism to oncogenesis and drug resistance [21,22,23]. We observed that among the tumor suppressors down-regulated in Dpep-resistant cells, a large majority have been described as hypomethylated in a variety of cancers and that most of these are subject to up-regulation by decitabine. Our findings thus support use of a hypomethylating agent such as decitabine as a cotreatment with Dpep in WT and resistant cancer cells and supply a potential rationale for their synergistic interaction.

Limitations of the study and future directions. There are a several limitations in the current study, which call for additional work in the future. For one, we examined only combinations of Dpep with ABT-263 and decitabine. There are other drugs that target pro-survival family members as well as alternative hypomethylating agents that remain to be assessed for efficacy in combination with Dpep. Second, we have focused on efficacy and have not delved deeply into the molecular mechanisms by which the various combinations assessed here synergistically affect cell survival. Pursuit of this area would have the potential to inform of additional as well as of more effective partners for combination with Dpep. Third, we generated and examined a limited number of Dpep-resistant cell lines and we compared gene regulation in only a single pair of responsive/resistant lines. Studies on additional such lines would provide a broader view of the basis of Dpep resistance and how it might be overcome. Fourth, available resources limited our animal studies to a single tumor type and to assessment of a single combination treatment. Further experiments along these lines would inform us about which combinations are the most promising and the degree to which they hold for various tumor types. Fifth, we have employed a cell-line-derived xenograft model in nude mice. Such models have potential well-recognized limitations in recapitulating human malignancies. In particular, while they do reveal drug-induced anti-tumor effects, they do not have the heterogeneity found in endogenous tumors and lack the appropriate tumor microenvironment and full host immune responses. Future studies of this type should use multiple orthotopic models employing PDX material and syngeneic models with fully intact immune systems. Sixth, we have not assessed cells freshly derived from patient tumors either in culture or in animal models. Such studies would indicate the efficacy of our proposed treatments on heterogeneous tumor material originating from multiple patients. Seventh, our study on the effect of decitabine and ABT-263 on Dpep-promoted sensitization of HCT116 cells to killing by NK-92MI cells is limited to a single cell line and does not include experiments on the molecular mechanisms underlying these responses. In addition, it would be of significant interest to determine whether such sensitization may occur in vivo with either NK-92MI cells or endogenous NK cells.

## 5. Conclusions

Cell-penetrating peptides like Dpep that target ATF5, CEBPB and CEBPD hold substantial promise for treatment of a variety of solid and liquid tumor types. Appropriate combinations with BCL2 family inhibitors and/or hypomethylating agents (represented here by ABT-263 and decitabine) show synergistic suppression of tumor cell growth/survival in vitro and in vivo. These observations support the potential for translating such combinations into therapeutic use.

## Data Availability

Raw and processed sequencing data from the referenced RNA-seq study [14] are available in the Gene Expression Omnibus under accession number GSE244579. Raw data from the current study are given in Supplementary File S2.

## Supplementary Materials

The following supporting information can be downloaded at: https://www.mdpi.com/article/10.3390/cells15090826/s1, File S1: Comparative analysis of transcriptomes of A375 Dpep-resistant#1 and A375 Dpep-responsive#1 cells exposed to Dpep for 48 h. File S2: Raw data from the current study. Figure S1. Pretreatment of HCT116 cells Dpep combined with either decitabine or ABT-263 synergistically sensitizes them to the killing actions of NK-92MI cells.

## Abbreviations

The following abbreviations are used in this manuscript:

WT	Wild type
CML	Chronic myeloid leukemia
AML	Acute myeloid leukemia
DEGs	Differentially expressed genes
NK	Natural killer cells
