# Supplementary material for: Dpep, a Cell-Penetrating Peptide Targeting ATF5, CEBPB and CEBPD, Synergistically Combines with ABT-263 and Decitabine to Inhibit Cancer Cell Growth and Overcome Dpep Resistance
Source: Cells. 2026 May 1;15(9):826. doi: 10.3390/cells15090826 (PMC13162579; doi:10.3390/cells15090826)
Supplement: Supplementary file 1 [file cells-15-00826-s001.zip › Supplementary Figure S1 and legend.pdf]

Supplementary Figure S1

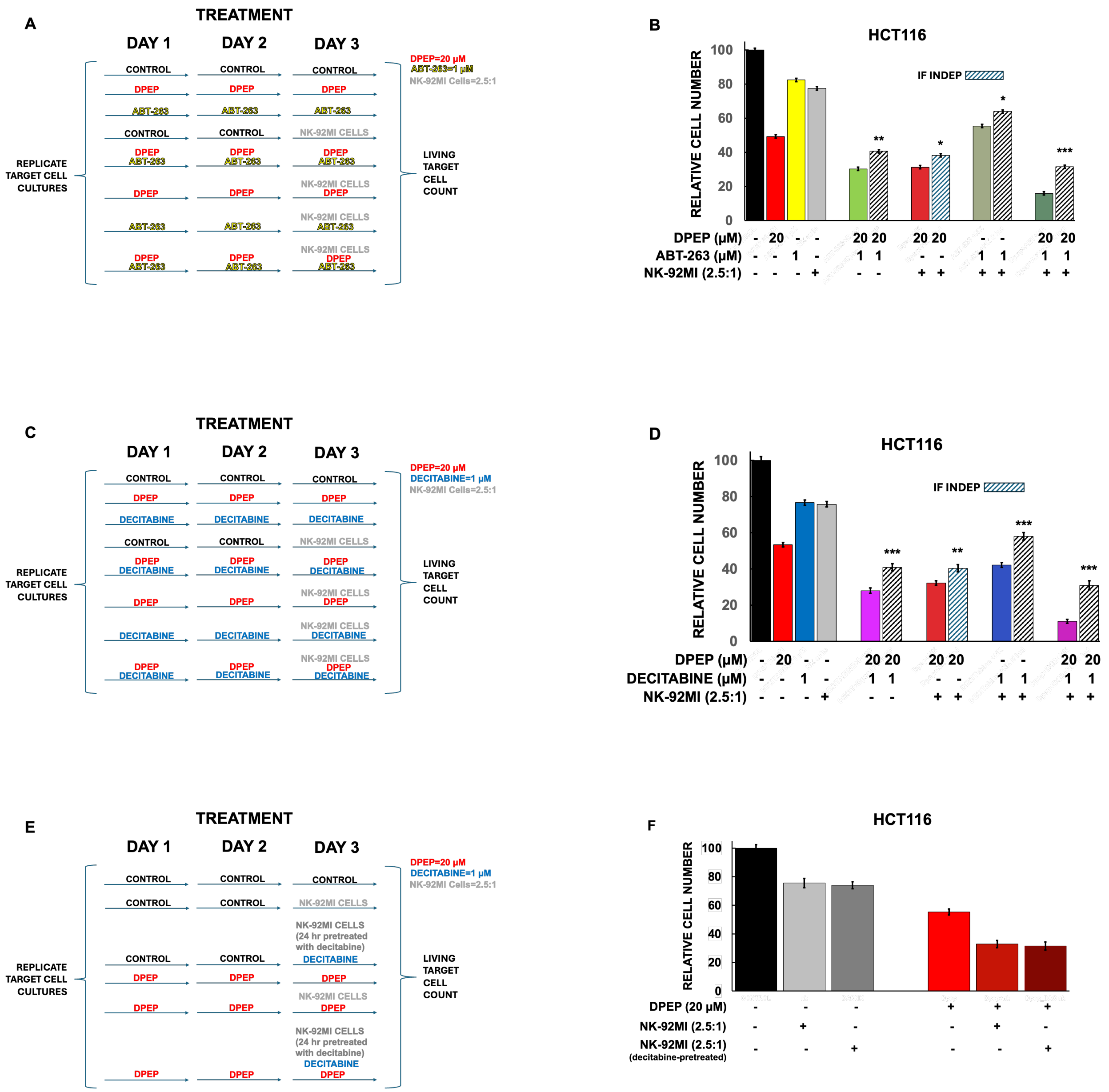

Supplementary Figure S1. Pretreatment of HCT116 cells Dpep combined with either decitabine or ABT-263 synergistically sensitizes them to the killing actions of NK-92MI cells. (**A**) Experimental scheme for pre-treatment of HCT116 cells with Dpep and/or ABT-263 prior to 24 hours exposure to NK-92MI cells. (**B**) Relative survival of HCT116 cells after pre-treatment with Dpep and/or ABT-263 followed by exposure to NK-92MI cells as described in

panel A. N=6 for all treatment conditions. (**C**) Experimental scheme for pre-treatment of HCT116 cells with Dpep and/or decitabine prior to 24 hours exposure to NK-92MI cells. (**D**) Relative survival of HCT116 cells after pre-treatment with Dpep and/or decitabine followed by exposure to NK-92MI cells as described in panel C. N=6 for all treatment conditions. (**E**) Experimental scheme for pre-treatment of NK-92MI cells with or without decitabine prior to exposure to HCT116 cells with or without pre-treatment with Dpep. (**F**) Relative survival of HCT116 cells pre-treated with or without decitabine followed by exposure to NK-92MI cells with or without decitabine pre-treatment as described in panel E. N=3 for all treatment conditions. All data are given as means±SEM. * p<0.0.05; ** p<0.005; *** p<0.0005.
